# Decoding circRNA translation: challenges and advances in computational method development

**DOI:** 10.3389/fgene.2025.1654305

**Published:** 2025-07-29

**Authors:** Jingjing Zhang, Rui Zhou, Huiling Zhang, Yin Peng, Jintao Meng, Wenhui Xi, Yanjie Wei

**Affiliations:** ^1^Shenzhen Institutes of Advanced Technology, Chinese Academy of Sciences, Shenzhen, China; ^2^ University of Chinese Academy of Sciences, Beijing, China; ^3^ College of Mathematics and Information, South China Agriculture University, Guangzhou, China; ^4^ Department of Pathology, Shenzhen University School of Medicine, Shenzhen, Guangdong, China

**Keywords:** circRNA, translation, bioinformatics, function, coding potential

## Abstract

In recent years, numerous studies have demonstrated that circRNAs play crucial biological roles through their capacity to encode functional proteins. Computational methods have become essential for investigating circRNA translation. In this review, we first outline circRNA biogenesis and translation mechanisms to establish the rationale for developing specialized computational strategies. We then summarize experimental techniques and existing databases that support computational method development. Subsequently, we provide a systematic introduction to existing circRNA translation analysis tools and their underlying algorithms, with emphasis on benchmarking the performance of sequence-based methods using a unified dataset. Our benchmarking revealed that: (1) cirCodAn achieved superior predictive accuracy while maintaining user accessibility; (2) the training data selection during method development critically impacts model performance. This review serves as a comprehensive reference for the selection and application of circRNA translation analysis methods and provides foundational guidance for the development and refinement of future computational tools.

## 1 Introduction

Circular RNAs (circRNAs) are covalently closed RNA molecules generated through back-splicing. They originate from diverse genomic regions, including exonic, intronic, and intergenic loci (Conn et al., 2024). Compared to linear mRNAs, circRNAs exhibit greater exonuclease resistance, extended half-lives, and lower expression levels (Jeck et al., 2013). Due to their low abundance and circular structure, circRNAs were initially regarded as aberrant splicing byproducts or non-functional ‘splicing noise’. However, circRNAs are now recognized as ubiquitous regulators (Liu et al., 2017). Recent advances reveal their conservation across species and functional roles in metabolic pathways and disease processes in both animals and plants (Ivanov et al., 2015; Zhang and Dai, 2022). The most well-documented function involves acting as miRNA sponges to post-transcriptionally regulate gene expression (Zhang et al., 2018; Zhang X. et al., 2020; Hashemi et al., 2025; Marei et al., 2025). Additionally, circRNAs interact with RNA-binding proteins (RBPs), modulate gene expression through RNA-RNA interactions, and can undergo cap-independent translation to produce functional peptides or proteins with biological activity (Zhou et al., 2020; Hossain et al., 2023; Yi et al., 2024).

Compared to proteins translated from linear mRNAs, circRNA-derived proteins are generally shorter (typically 100–500 amino acids) yet play significant roles in development, disease progression, and tumorigenesis (Peng et al., 2021; Wei et al., 2025). For instance, Peng et al. identified the functional peptide GSPT1-238aa encoded by circGSPT1, which interacts with the vimentin/Beclin1/14-3-3 complex and regulates autophagy through the PI3K/AKT/mTOR signaling pathway in gastric cancer cells (Hu et al., 2022). Beyond disease regulation, circRNAs are increasingly recognized as a promising platform for vaccine development (Zhang et al., 2024), with most circRNA-based vaccines designed around their encoded therapeutic peptides/proteins (Chen et al., 2023). As vaccine vectors, circRNAs achieve higher expression at lower doses than linear mRNA counterparts, underscoring their therapeutic potential. Hence, Further investigation into circRNA translation functions and applications is warranted (Lokras et al., 2024).

Due to their covalently closed structure, circRNAs lack 5′ caps and 3′ poly(A) tails, resulting in a translation initiation mechanism distinct from linear mRNA (Hwang and Kim, 2024). Canonical mRNA translation employs the 5′ m^7^G cap structure and initiation factors—including eIF4E, eIF4G, and eIF4A—to recruit ribosomes (Gentry et al., 2025). In contrast, circRNAs rely cap-independent mechanisms, including: internal ribosome entry site (IRES)-mediated translation, N^6^-methyladenosine (m^6^A)-mediated initiation (also referred to as MIRES), and other forms of internal initiation (Nott et al., 2004; Yang and Wang, 2019; Zhang G. et al., 2023). These pathways employ diverse regulatory elements whose full functional repertoire remains incompletely characterized and warrants further investigation.

Advances in circRNA functional research are critically dependent on bioinformatics tools (Drula et al., 2024). Diverse computational methodologies have been applied to circRNA prediction and functional characterization, including detection tools (e.g., CIRI2) (Gao et al., 2018), sequence assembly tools (e.g., JCcirc) (Zhang J. et al., 2023), function prediction tools (e.g., iCRBP-LKHA, MSMCDA, CircDA) (Niu et al., 2024; Yuan et al., 2024; Zhang et al., 2025). For translation-specific prediction, specialized algorithms have emerged. Experimental evidence-based approaches integrate high-throughput data—such as ribosome profiling (Ribo-seq) and mass spectrometry (MS)—to validate translation events. Representative tools include MStoCIRC (Cao and Li, 2022), CircCode (Sun and Li, 2019), and CircPro(Meng et al., 2017). Complementarily, sequence-driven methods leverage machine learning and deep learning frameworks to predict translational potential from intrinsic circRNA features, exemplified by cirCodAn and CICADA (Barbosa et al., 2024; Fan et al., 2025). In conclusion, these methodologies enable a systematic and accurate assessment of circRNA translation potential.

**FIGURE 1:**
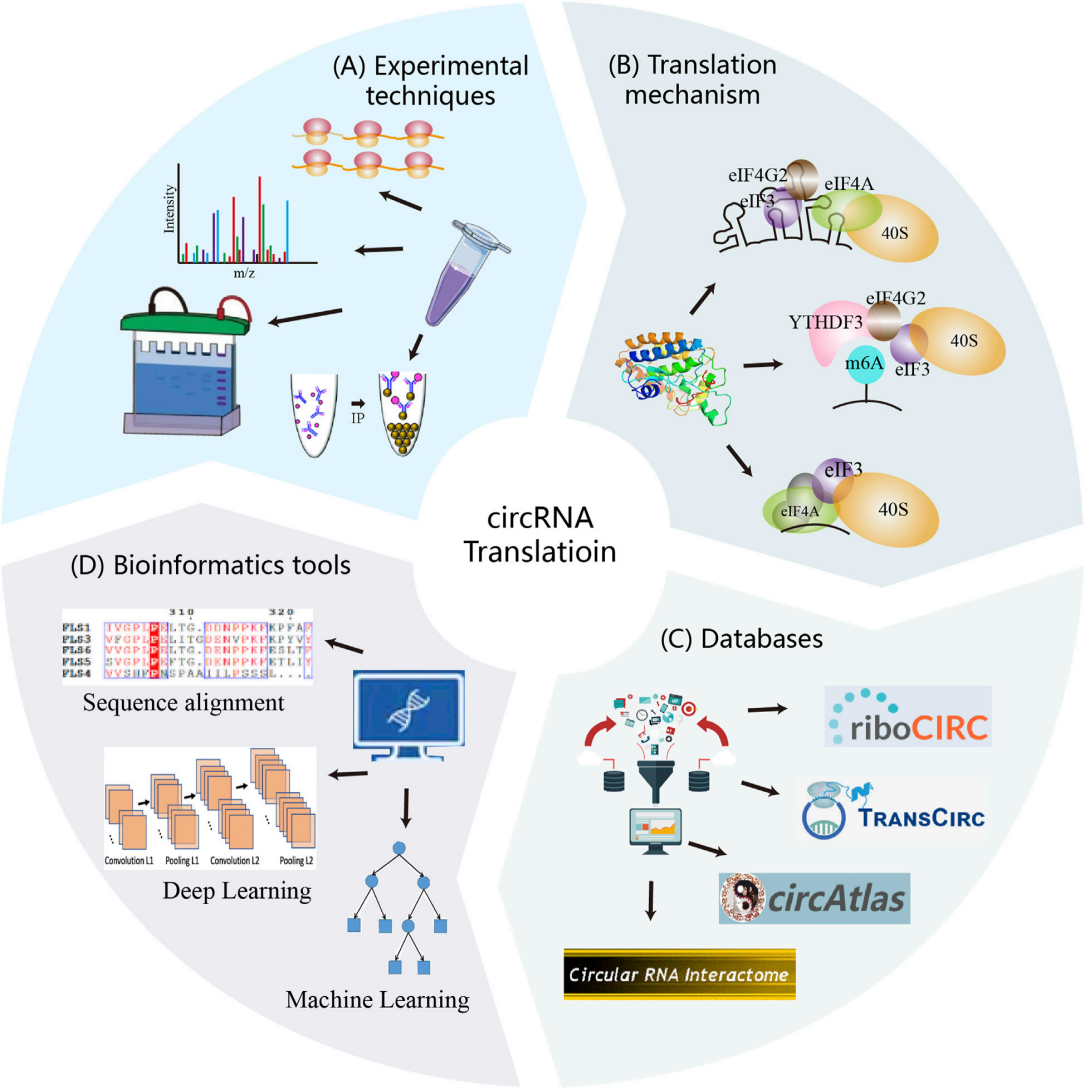
Experimental techniques, translation mechanisms, databases, and computational strategies constitute the core components of circRNA translation research. These elements interact synergistically to advance the field. Experimental methods uncover translation mechanisms and validate circRNA-derived proteins, generating reliable data. Mechanism provide the theoretical foundation for computational strategy development. Both experimental and computational data are deposited into databases, which serve as resources for researchers and support further method development. In turn, computational analyses guide experimental validation and contribute newly predicted data to the databases.

In this review, we systematically introduce the information related to circRNA translation, as shown in Figure 1. First, we outline circRNA biogenesis and translation mechanisms, establishing the theoretical foundation and necessity for specialized analysis strategies. We then summarized experimental techniques and databases supporting computational methodology development. Building on this foundation, we systematically reviewed existing circRNA translation analysis tools and their underlying strategies, with particular emphasis on practical applicability across biological contexts. Finally, we benchmark sequence-based methods using a unified dataset and propose actionable directions for future methodology development in circRNA translation prediction.

## 2 The biogenesis and translation mechanism of circRNAs

### 2.1 Molecular mechanisms of circRNAs biogenesis

**FIGURE 2:**
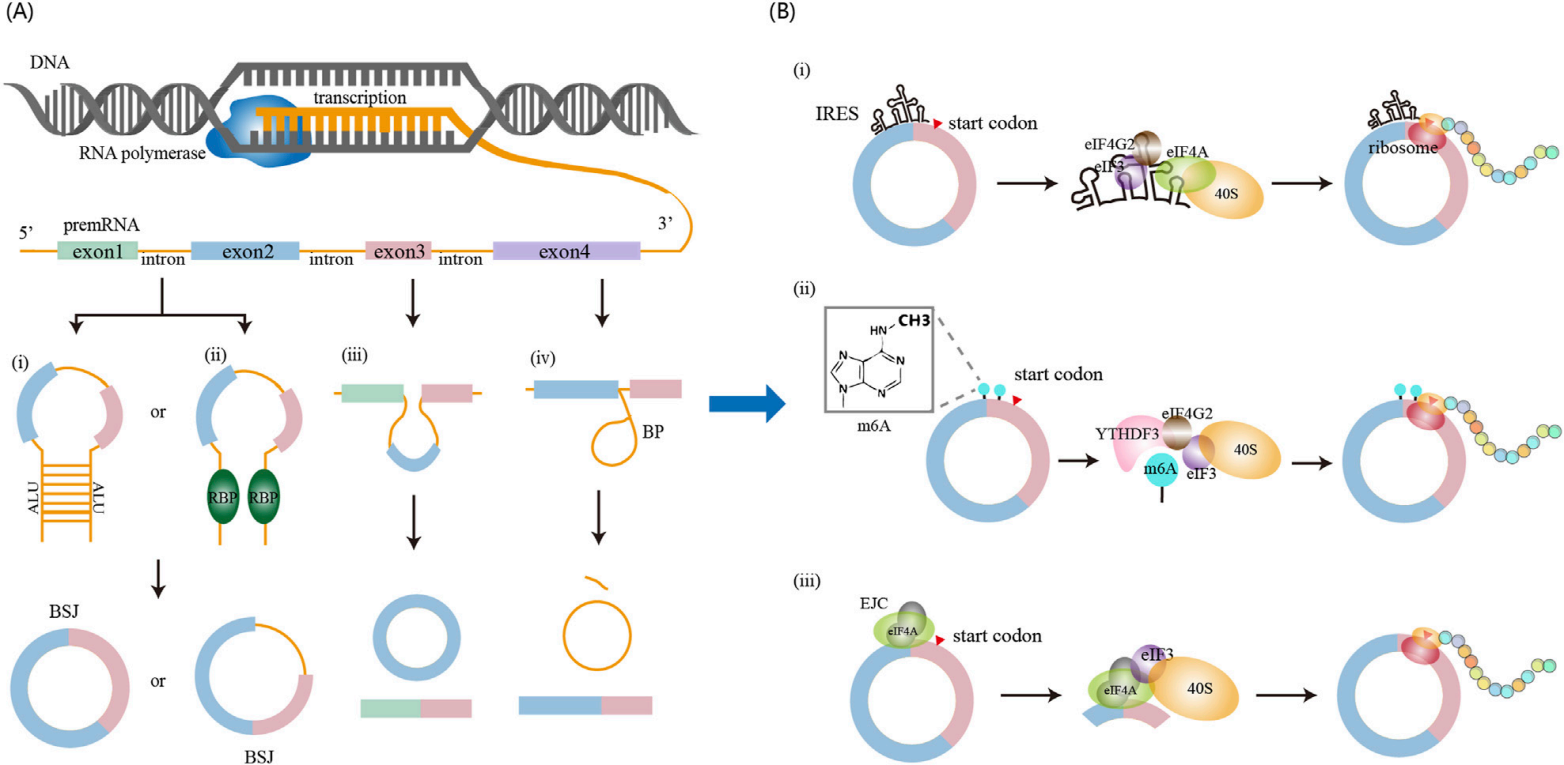
circRNA biogenesis and translation mechanisms. **(A)** circRNA biogenesis occurs in the nucleus and can be promoted through four major pathways: (i) intron pairing-driven circularization, (ii) RNA-binding protein (RBP)-mediated circularization, (iii) exon skipping-driven circularization, and (iv) lariat-driven circularization. **(B)** circRNA translation occurs in the cytoplasm and proceeds via cap-independent mechanisms, including: (i) IRES-mediated initiation, (ii) m^6^A-mediated initiation, and (iii) EJC-mediated initiation.

The defining feature of circRNAs is their covalently closed circular structure, formed through back-splicing—a regulated splicing variant where a downstream splice donor covalently joins an upstream splice acceptor via a 3′-5′ phosphodiester bond, generating a continuous RNA circle (Kristensen et al., 2019). This splicing mechanism provides the theoretical basis for the identification of circRNAs from high-throughput sequencing data. As illustrated in Figure 2A, circRNAs biogenesis occurs through four mechanisms.

(i) Intron pairing-driven circularization. Dubin et al. provided first evidence that long inverted repeats (IRs) flanking the mouse *Sry* gene promote circSry formation in cultured cells (Dubin et al., 1995). Subsequently, genome-wide analyses identified enrichment of Alu repeats (a subclass of inverted repeats) in introns adjacent to back-splice sites, establishing complementary sequence pairing as a fundamental circRNA biogenesis mechanism (Jeck et al., 2013). Notably, paired Alu elements flanking circularized exons show ∼5-fold enrichment at human exon circularization sites compared to linear splicing controls (Jeck et al., 2013). This principle of cis-regulatory complementarity has been effectively utilized in synthetic circRNA engineering, where flanking complementary sequences dramatically enhance circularization efficiency (Wesselhoeft et al., 2018).
(ii) RNA-binding-protein-driven circulatization. Ashwal-Fluss et al. first identified that the splicing factor muscleblind (*MBL*) in *Drosophila* and its vertebrate homolog, muscleblind-like protein 1 (MBNL1), promote exon circularization (Ashwal-Fluss et al., 2014). Among MBL isoforms, only MBL-C (the MBL predominant isoform in fly heads) and MBL-A enhance circularization. Furthermore, both *Drosophila* MBL-C and MBL-A isoforms promote circularization at the human *MBNL1* locus, indicating evolutionary conservation of this mechanism (Ashwal-Fluss et al., 2014). Beyond MBL, multiple RBPs facilitate exon circularization, including adenosine deaminases acting on RNA (ADAR), quaking (QKI), FUS, ATP-dependent RNA helicase A (DHX9), and SR-rich proteins (Conn et al., 2015; Ivanov et al., 2015; Kramer et al., 2015; Aktaş et al., 2017).
(iii) Exon-skipping-driven circularization. During alternative splicing, pre-mRNA can generate both a linear mRNA transcript and a lariat structure containing skipped exons. The skipped exons within the lariat are subsequently subjected to back-splicing, and circRNA formation may occur. Notably, the exons incorporated into the resulting circRNA are distinct from those present in the linear mRNA transcript, as each originates from separate splicing events within. This mechanism was first identified by Zaphiropoulos, who found exon circularization in the rat cytochrome P-450 2C24 gene (Zaphiropoulos, 1997). Subsequent studies in human and mouse models revealed similar exon skipping–mediated circularization events across multiple genes, indicating that this pathway represents a widespread and conserved mechanism of circRNA biogenesis (Surono et al., 1999; Kelly et al., 2015).
(iv) Lariat-driven circularization. Pre-mRNA splicing involves the removal of introns and the ligation of exons. During this process, intron excision generates a lariat structure (Padgett et al., 1984), a transient intermediate generated through a 2′-5′ phosphodiester bond between the 5′ splice site and the branch point, with a free 3′ tail (Keller, 1984; Yoshimoto et al., 2009; Borao et al., 2021). Typically, these lariats are rapidly degraded by debranching enzymes and exonucleases (Mohanta and Chakrabarti, 2021). However, it has been hypothesized that some lariat structures can evade degradation and serve as precursors for intron-derived circRNAs(Zhang et al., 2013; Talhouarne and Gall, 2014). Emerging evidence indicates that lariat-derived circRNAs also play functional roles in animals (Stoll et al., 2020; Robic et al., 2021).

### 2.2 Molecular mechanisms of circRNA translation

For many years, circRNAs were regarded as non-coding RNAs. However, emerging evidence now demonstrates that a subset of circRNAs bind to polysomes and undergo translation. Unlike linear mRNAs, circRNAs lack a 5′ cap structure and instead initiate translation through cap-independent pathways (Figure 2B).

(i) Internal initiation of translation mediated by IRESs. IRESs are *cis*-acting RNA elements that facilitate cap-independent translation by directly recruiting ribosomes to internal regions of transcripts (Yang and Wang, 2019). As understanding of circRNA function has advanced, functional IRES elements have been identified within circRNA sequences, enabling their translation initiation (O'Leary et al., 2025). Canonical IRESs function as structural scaffolds recognized by eIF4G2 and the eIF3 complex, which collaborates with eIF4A. This facilitates recruitment of the 40S ribosomal subunit and assembly of the 43S pre-initiation complex to initiate translation (Chen and Sarnow, 1995; Roberts and Wieden, 2018; Shi et al., 2020). Moreover, IRES activity is modulated by IRES *trans*-acting factors (ITAFs), including heterogeneous nuclear ribonucleoprotein (hnRNP) family (e.g., hnRNPI, hnRNPQ, hnRNPU), as well as PABPC1 and QKI. These ITAFs enhance circRNA translation efficiency by stabilizing IRES structures or inducing RNA conformational changes (Kafasla et al., 2009; Romanelli et al., 2013; Ashwal-Fluss et al., 2014; Godet et al., 2019; Fan et al., 2022). Unbiased screening using cell-based reporter system has also revealed that AU-rich motifs (∼10 nucleotides) exhibit IRES-like activity capable of initiating circRNA translation (Wang, 2018; Yang and Wang, 2019). Given the widespread occurrence of such elements in circRNAs, IRES-mediated cap-independent translation of circRNAs is likely a prevalent and functionally significant mechanism.
(ii) Internal initiation of translation mediated by m^6^A modification. Early studies demonstrated that m^6^A methylation enables cap-independent translation of linear mRNAs under cellular stress conditions (Meyer et al., 2015). Subsequent work revealed that circRNAs are also methylated by the METTL3/METTL14 complex, with m^6^A modification influencing multiple aspects of circRNA biology, including translation regulation, decay kinetics, and innate immune modulation (Zhang L. et al., 2020). In the context of translation, m^6^A-modified circRNAs recruit the reader protein YTHDF3, which facilitates assembly of the translation initiation machinery, notably through eIF4G2 recruitment, to drive protein synthesis. Yang et al. further showed that circRNA translation is promoted by the METTL3/14 methyltransferase complex and suppressed by the demethylase FTO (Yang et al., 2017). Moreover, short RNA motifs containing m^6^A-induced ribosome engagement sites have been identified as IRES-like elements capable of driving circRNA translation (Huang et al., 2021). These findings suggest an interplay between canonical IRES elements and epitranscriptomic mechanisms in cap-independent translation. To fully elucidate the mechanisms underlying circRNA translation, further comprehensive studies integrating IRES, m^6^A, and other regulatory elements are warranted.
(iii) Internal initiation of translation mediated by the exon junction complex (EJC). The EJC, a multi-protein complex critical for circRNA biogenesis, includes eIF4A3 as one of its core components (Zhang Y. et al., 2023). Recent studies demonstrate that the EJC directly facilitates internal initiation of circRNA translation (Chang et al., 2023; Lin et al., 2023; Xiong et al., 2023). Following its deposition on circRNAs, the EJC recruits the eIF3 complex via interactions between eIF4A3 and the eIF3G subunit (Chang et al., 2023). This suggests that the EJC functions as a molecular scaffold that bridges the eIF3 complex and the 40S ribosomal subunit to circRNAs, enabling cap-independent translation initiation. Moreover, eIF4A3 within the EJC context promotes internal translation initiation in an eIF3-dependent manner, and this ability is further enhanced by other components of the EJC. Collectively, these findings indicate that both EJC complexes deposited on circRNAs during back-splicing and free eIF4A3 molecules on circRNAs can facilitate ribosome recruitment and translation initiation.

In summary, circRNAs exhibit distinct structural properties and employ cap-independent translation mechanisms. Therefore, applying translation analysis methods designed for linear RNAs is likely to yield inaccurate assessments of circRNA coding potential. To address this limitation, dedicated computational approaches must be developed that account for the unique sequence architecture and translation mechanisms of circRNAs. Such specialized tools will provide essential guidance for the functional characterization of circRNA-encoded proteins.

## 3 Experimental strategies for analyzing circRNA translation

Experimental strategies form the cornerstone of circRNA research. For studying circRNA translation, these methods are essential to determine coding capacity and elucidate initiation mechanisms. The resulting data provide critical support for developing computational methods to predict translatable circRNAs. This section describes two categories of experimental approaches for studying circRNA translation (Table 1): high-throughput screening for genome-wide identification of translated circRNAs, and targeted validation for mechanistic investigation of individual circRNAs.

**TABLE 1:** Overview of experimental approaches applied in circRNA translation research.

Method	Throughput	Advantages	Disadvantages
Ribosome profiling	High	Transcriptome-wide analysis; Ribosome occupancy and dynamics profiling.	Indirect evidence; Lacks confirmation of protein expression; Risk of false positives.
Polysomal fractionation	High	Established technique; High reliability through combined transcriptomic and quantitative analysis.	Limited resolution; Unable to localize translation start sites; Risk of false positives.
Mass spectrometry	High	High sensitivity; Direct evidence of translation; Proteome-wide analysis.	Limited sensitivity for low-abundance peptides; Database-dependent.
Western blot	Low	High specificity; Intuitive results.	Low throughput; Antibody design complexity.
Immunoprecipitation-mass spectrometry	Low	High sensitivity and specificity; Enrichment of low-abundance proteins.	Antibody dependence; High cost; Risk of false positives.

### 3.1 High-throughput techniques

(i) Ribosome profiling is a high-throughput technique that captures ribosome-protected fragments (RPFs) from actively translating RNA molecules (Ingolia et al., 2009). In this approach, RNase digestion degrades single-stranded RNA regions that are not protected by ribosomes, generating ∼28–30 nucleotide RPFs for sequencing. These fragments are derived from all translated RNAs, both linear and circular RNAs. Detection of RPFs spanning a circRNA’s BSJ is a hallmark of its translational potential (Sun and Li, 2019). However, ribosome association alone does not confirm functional protein production (Guttman et al., 2013), as Ribo-seq signals may include nonproductive translation events. Thus, additional experimental validation—such as MS or Western blotting—is essential to verify protein expression.
(ii) Polysomal fractionation separates translational complexes via sucrose density gradient centrifugation, fractionating RNAs based on their ribosomal engagement (Rodriguez-Martinez and Young-Baird, 2025). RNAs co-sedimenting with multiple ribosomes—polysomes—typically reflect high translational activity. In this approach, RNA is extracted from different gradient fractions and subsequently sequenced to identify circRNAs enriched in polysome-associated fractions. Like ribosome profiling, this method captures both linear and circular RNAs; thus, BSJ analysis is required to confirm circRNA-specific translation. As ribosome binding alone does not guarantee productive translation, this technique may yield false-positive results and requires additional validation (MS or Western blotting). Compared to ribosome profiling, polysomal fractionation avoids specialized footprint library construction, offering a less technically demanding workflow. However, it exhibits lower sensitivity in detecting low-abundance circRNAs.
(iii) Mass spectrometry provides the most direct evidence for circRNA translation by identifying peptides derived from circRNA open reading frames (cORFs) (Fang et al., 2022). In this proteomic method, total cellular proteins are extracted and digested into peptides, which are analyzed by MS. Translation is confirmed when peptides uniquely map to the BSJ region. MS enables proteome-scale screening for circRNA-derived peptides but faces sensitivity limitations in detecting low-abundance species due to ion suppression and dynamic range constraints.

### 3.2 Verification techniques for single circRNA

Although high-throughput techniques like ribosome profiling and MS offer transcriptome- and proteome-wide perspectives on circRNA translation, validating the coding capacity of specific circRNAs requires targeted experimental approaches. These methods distinguish circRNA-encoded proteins from products of homologous linear transcripts.

Western blotting verifies circRNA-derived proteins using custom antibodies against BSJ-spanning epitopes, unique molecular signatures enabling discrimination from linear homologous linear isoform products (Song et al., 2023). Immunoprecipitation coupled with mass spectrometry (IP-MS) offers a highly sensitive method for validating circRNA-encoded proteins (Zang et al., 2024). This method employs antibodies targeting circRNA-specific epitopes to enrich protein, followed by high-resolution MS identification of BSJ-spanning peptides. Identification of these junctional peptides provides presumptive evidence of circRNA-derived translation. Despite its sensitivity and specificity, the IP-MS approach depends heavily on antibody specificity and requires substantial technical expertise. Collectively, these targeted strategies complement bioinformatic and omics methods for confirming individual circRNA translatability.

## 4 Databases supporting circRNA translation research

Growing interest in functional circRNA analysis has established translation studies as a key focus, driving the accumulation of diverse translation evidence. Consequently, this information is now integrated into major circRNA databases, including specialized resources dedicated to circRNA translation. This outlines the core data types, features, and applications of these repositories. As cataloged in Table 2, circRNA translation-related databases are categorized as: general circRNA databases and specialized circRNA translation databases. These resources support mechanistic research while providing valuable datasets for the development, training, benchmarking, and validation of computational tools designed for circRNA translation analysis.

(i) CircRNA translation databases curate evidence related to the coding potential of circRNAs. These databases typically provide core details such as genomic loci and host genes, though they often lack comprehensive functional characterization. riboCIRC and TransCirc are two databases integrating computational predictions and experimental evidence to investigate circRNA translation (Huang et al., 2021; Li et al., 2021).

**TABLE 2:** Databases publicly available for circRNA translation research.

Database	Class	Description about translation	URL	Update
CircInteractome	Comprehensive	IRES elements in circRNA sequences were annotated based on the IRESite database	https://circinteractome.nia.nih.gov/	2020
C2CDB	Comprehensive	Translation-related elements in circRNA sequences were annotated using data from the IRESbase and m6A-Atlas databases, with all putative ORFs predicted by custom in-house scripts	http://pengyonglab.com/c2cdb/	2024
circRNADb	Comprehensive	The VIPS tool was used to predict IRES elements within each circRNA sequence, identify the longest ORFs, and characterize the corresponding translated proteins	http://reprod.njmu.edu.cn/circrnadb	2024
circAtlas 3.0	Comprehensive	IRESfinder and ORFfinder were applied to predict IRES elements and coding peptides in circRNA. sequences	https://ngdc.cncb.ac.cn/circatlas	2023
riboCIRC	Translation	Translatable circRNAs were categorized into two groups: those predicted through Ribo-Seq data analysis and those experimentally validated and curated from the literature, with annotated positions of IRES and m6A modifications	http://ribocirc.com/index.html	2021
TransCirc	Translation	Multiple complementary methods were integrated to analyze translation elements and comprehensively evaluate the translational potential of circRNAs, including ORFs, IRES, m6A modifications, Ribo-Seq data, MS data, translation initiation sites, and machine learning-based predictions of translation probability	https://www.biosino.org/transcirc/	2021

riboCIRC specializes in systematically identifying ribosome-associated circRNAs from public Ribo-seq datasets. It also predicts circRNA-specific ORFs and annotates key translation initiation elements, including IRESs and m^6^A modification sites. Furthermore, riboCIRC integrates evidence from MS and provides a manually curated catalog of experimentally validated translatable circRNAs from the literature. TransCirc aggregates seven types of evidence to infer circRNA coding potential. These include: (1) ribosome and polysome profiling data; (2) experimentally mapped translation initiation sites (TISs); (3) IRES elements; (4) m^6^A modifications; (5) circRNA-specific ORFs length; (6) machine learning-derived sequence composition scores; and (7) direct MS detection of peptides spanning back-splice junctions. By integrating the direct and indirect lines of evidence, TransCirc provides a comprehensive platform for evaluating translation capacity and putative products of human circRNAs.

(ii) Comprehensive circRNA databases systematically curate extensive information on circRNAs, encompassing basic annotations such as genomic coordinates, nucleotide sequences, and host genes, as well as functional annotations (e.g., miRNA/RBP binding sites), and coding potential assessment.

circRNADb (Chen et al., 2016) offers comprehensive circRNA annotations and evaluates coding potential through multiple approaches. It predicts IRES elements using the VIPS algorithm and identifies ORFs exceeding 300 nt. In addition, it annotates functional protein domains and post-translational modification sites using SMART and other established tools. The database also integrates known human proteins (UniProt 2013_07) with circRNA-encoded sequences and validates peptides through MS data from PRIDE. CircInteractome integrates publicly available circRNA, miRNA, and RBP datasets to facilitate the bioinformatic analyses of circRNA binding sites and their potential regulatory roles (Panda et al., 2018). In the context of circRNA translation, researchers use experimentally validated IRES sequences from the IRESite database to align with mature circRNA sequences to identify putative IRES within circRNAs. Another representative resource, C2CDB, provides basic annotations for circRNAs, including sequence and expression profiles, and incorporates functional features such as miRNA and RBP interactions, predicted coding potential, and secondary structure information (Zuo et al., 2024). To assess translational potential, circRNA sequences are annotated with translational elements based on data from IRESbase and m6A-Atlas, and putative ORFs are predicted with in-house algorithms. Moreover, C2CDB integrates publicly available translatome datasets to provide *in vivo* evidence of circRNA translation. circAtlas 3.0 is another large-scale resource that collected circRNA data from 33 tissue types across 10 vertebrate species (Wu et al., 2024). To explore the coding capacity of circRNAs, IRESfinder and ORFfinder are employed to identify potential IRES elements and coding sequences within circRNAs.

## 5 Computational methods for circRNA translation

Computational tools for predicting circRNA translation are indispensable for advancing circRNA functional studies. In recent years, numerous bioinformatics methods have been developed to assess the translational potential of circRNAs, including both novel circRNA-specific algorithms and linear RNA predictors adapted for circRNAs. To systematically categorize these methods, we classify them into two groups: (1) Translational Regulatory Element Predictors, (2) Integrated circRNA Translation Potential Predictors.

### 5.1 Translational regulatory element predictors

The unique translation mechanisms of circRNA involve multiple elements influencing, including IRESs, m^6^A modifications, ORFs, and TISs. Corresponding computational tools have been developed to detect these elements. For example, ORFfinder (https://www.ncbi.nlm.nih.gov/orffinder/) identifies ORFs within nucleotide sequences, providing genomic coordinates and translated peptide sequences. IRESfinder employs machine learning with framed k-mer features to predict IRES elements, utilizing a logistic regression model trained on experimentally validated sequences (Zhao et al., 2018).

Recently, several specialized computational methods have been developed for analyzing translation-related elements in circRNAs. Representative tools include CircTIS, TransRM, and DeepCIP (Barbosa et al., 2023; Zhou et al., 2023; Liu et al., 2025). CircTIS predicts TISs in circRNAs, considering both canonical (AUG) and non-canonical, while accounting for circular topology. It employs a weighted degree string kernel to encode sequence fragments surrounding candidate TISs, with an SVM classifier estimating TIS probability. TransRM is the first computational model developed to predict the effects of base-resolution m^6^A modifications on circRNA translation. Its weakly supervised learning framework integrates CNNs and BiGRUs. Sequence features are extracted by two CNN layers, while m^6^A site features are encoded using word2vec. The BiGRU module estimates the contribution of each m^6^A site to translational potential, and a random forest classifier integrates these features and predicts the likelihood of circRNA translation. DeepCIP is a multi-model deep learning framework designed to predict circRNA IRESs by integrating both sequence and structural information. Sequence features, including one-hot encoding, nucleotide chemical properties (NCP), and dinucleotide physicochemical properties (DPCP), are learned using Sentence-State LSTM (S-LSTM), while secondary structural features are trained using GCN.

### 5.2 Integrated circRNA Translation Potential Predictors

#### 5.2.1 CircRNA-specific predictors

**FIGURE 3:**
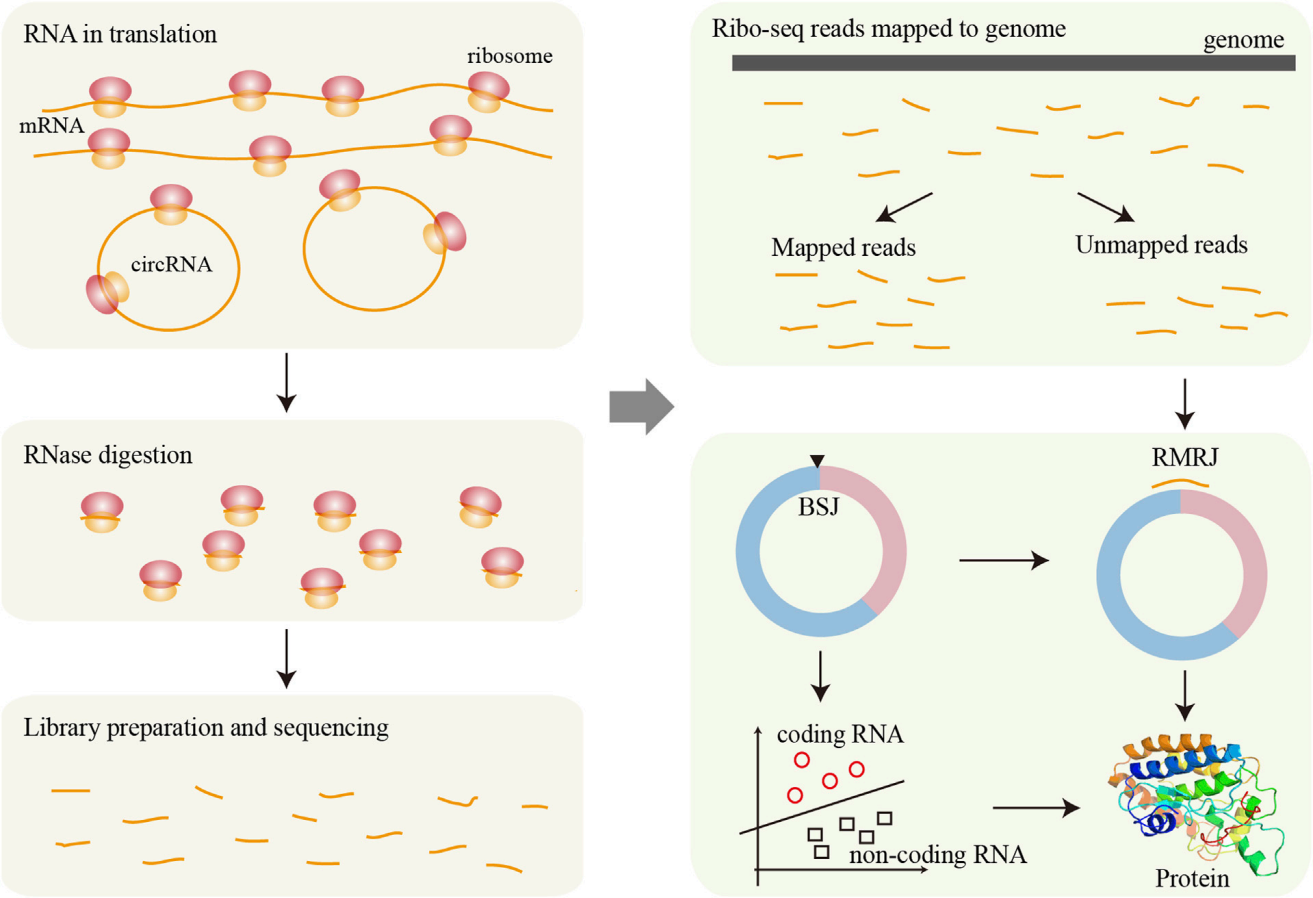
Framework for circRNA translation analysis based on Ribo-seq data. During translation, ribosomes bind to RNA sequences and associated regions from RNase digestion. After RNase treatment, the ribosome-protected fragments are isolated, used to construct sequencing libraries, and then sequenced. The resulting reads are mapped to the reference genome, and unmapped reads may originate from circRNAs. These candidate fragments are further screened to identify potential translation events derived from circRNAs. Finally, various machine learning methods can be applied to assess the translational potential of circRNAs in greater detail.

Ribo-Seq enables transcriptome-wide analysis of translated RNAs, serving as primary evidence for circRNA coding potential assessment. Computational tools leveraging Ribo-seq data follow a unified analytical framework (Figure 3). CircPro identifies protein-coding circRNAs by integrating total/poly(A)- RNA-Seq and Ribo-Seq data (Meng et al., 2017). It comprises three key modules: (1) circRNA detection using CIRI2 from SAM files; (2) coding potential evaluation via CPC, which provides classification, scores, and detailed ORF information; and (3) junction read identification from Ribo-Seq data. CircPro outputs predicted protein sequences in FASTA format. CircCode identifies circRNAs with coding potential using Ribo-Seq data (Sun and Li, 2019). Initially, it employs Trimmomatic, Bowtie, and STAR to preprocess and align raw Ribo-Seq reads to the reference genome. Unmapped reads are then mapped to circRNA sequences to detect RMRJ (Ribo-seq Read-Mapped Regions on Junctions). The mapped sequences in the circRNAs are classified into coding or noncoding circRNAs using BASiNET (J48 model). Finally, translated short peptides are identified as putative coding regions within circRNAs.

**FIGURE 4:**
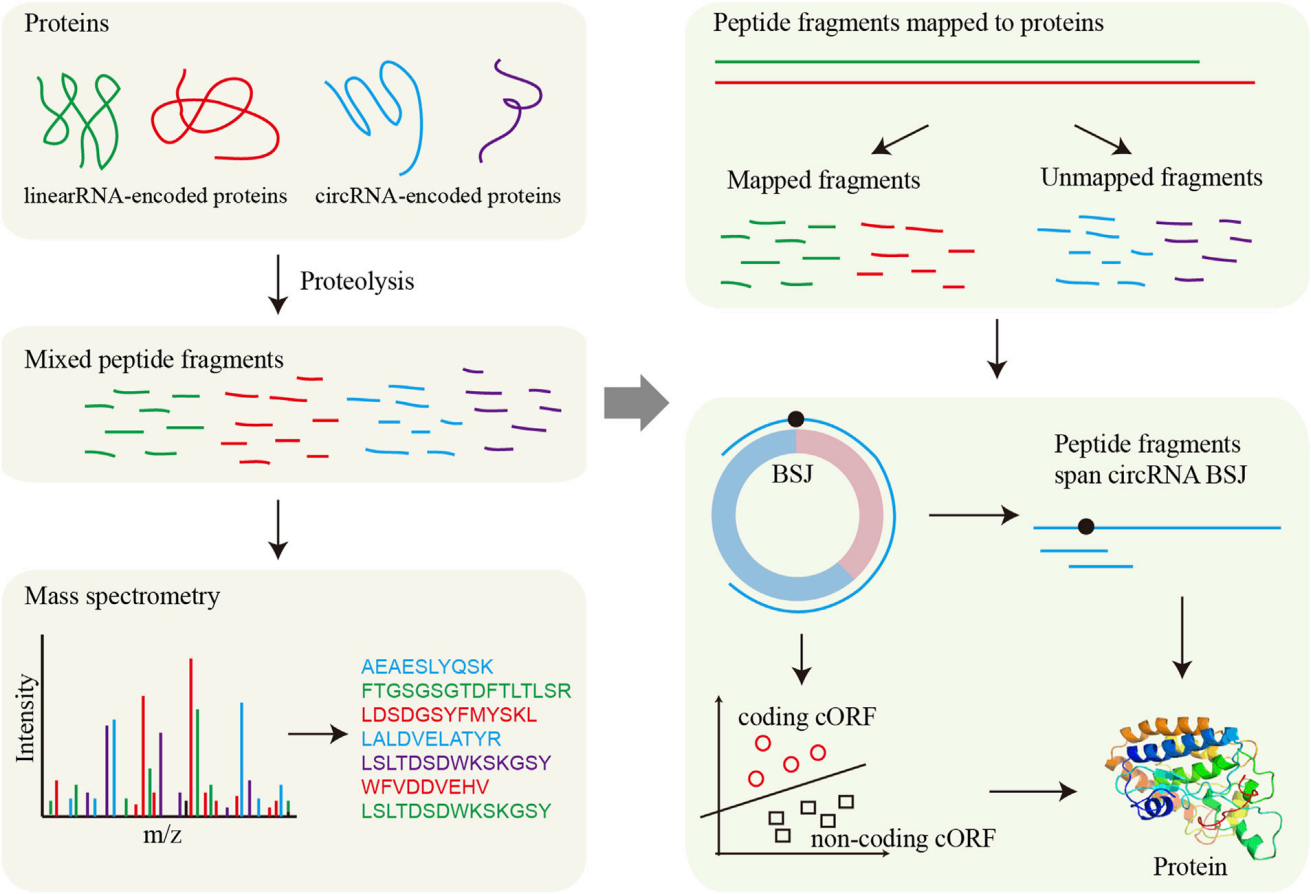
Framework for circRNA translation analysis based on MS. MS directly detects protein fragments (peptides), which are then aligned to reference protein sequences to determine their origin. For circRNAs, potential circRNA-encoded proteins are first predicted, and the presence of these proteins is validated by matching the detected fragments to the predicted circRNA-derived sequences.

MS-based proteomics provides direct detection of translated peptides, with its application to circRNA translation illustrated in Figure 4. MStoCIRC utilizes MS data to evaluate the translational potential of circRNAs (Cao and Li, 2022). The pipeline begins by translating the BSJ regions of circRNAs in all six reading frames to construct a reference peptide database. The raw MS data are then searched against this database to identify peptides potentially derived from circRNAs. Next, candidate cORFs are predicted, and their translational potential is assessed using a Naive Bayes classifier. The identified peptides are mapped to the predicted cORFs to determine if they span the BSJ, providing direct evidence of translation. Finally, overlapping peptides are merged to reconstruct the longest contiguous peptide sequences spanning the BSJ, offering strong support for circRNA translation.

Beyond Ribo-seq and MS-dependent approaches, sequence-based methods enable coding potential assessment of circRNAs without auxiliary experimental data. cirCodAn predicts circRNA coding sequences using its GHMM-circ architecture, a customized adaptation of the generalized hidden Markov model (GHMM) originally implemented in the CodAn tool for linear transcripts (Barbosa et al., 2024). GHMM-circ is specifically designed to capture the distinctive translation mechanism of circRNAs by modeling a transition from a non-coding state (NOCOD) through a defined start region into an extended coding state (CDS). This framework enables the accurate representation of the full circular translation process. CICADA evaluates circRNAs coding potential through multi-feature integration (Fan et al., 2025). It first identifies high-potential coding regions (HPCRs) by employing a sliding window strategy guided by the ANT scoring matrix, followed by a dynamic programming algorithm to determine the optimal HPCR. To build its predictive model, CICADA extracts a comprehensive set of features from both coding and non-coding circRNA datasets, including sequence composition, evolutionary conservation, secondary structure, and mechanistic indicators. These features are used to train a random forest classifier, which then performs binary classification to predict whether given circRNAs are likely to have coding potential. Additionally, CICADA also allows users to submit sequences and obtain prediction results (http://121.196.54.69:8002/CICADA/home).

CircPrimer 2.0 is a multi-evidence integration platform for comprehensive circRNAs annotation and coding potential assessment (Zhong and Feng, 2022). Beyond its core functions in circRNA identification and primer design, the tool incorporates three translational element predictors: ORFs, IRESs, and m^6^A modification sites. IRES elements are predicted using TGBoost models trained on global k-mer frequencies, while m^6^A modification sites are identified by mapping high-confidence sites from the m6A-Atlas database and detecting consensus DRACH/RRACH motifs directly through the circPrimer 2.0 interface.

#### 5.2.2 Linear RNA Predictors Adapted for circRNAs

In addition to methods specifically developed for circRNAs, several tools originally designed for linear RNAs—such as CPC2, CPAT, and CNCI—have also been applied in the early exploration of circRNA translation potential. CPC2 (Coding Potential Calculator 2) evaluates the protein-coding potential of transcripts using four intrinsic features: Fickett TESTCODE score, ORF length, ORF integrity, and isoelectric point (Kang et al., 2017). It employs an SVM classifier trained on these features and is available as both a user-friendly web server and a standalone package (http://cpc2.cbi.pku.edu.cn). CPAT (Coding-Potential Assessment Tool) is an alignment-free tool that distinguishes coding from non-coding transcripts using logistic regression. It incorporates four sequence-derived features: maximum ORF length, ORF coverage, Fickett TESTCODE score, and hexamer usage bias (Wang et al., 2013). CPAT also provides an intuitive web interface that allows rapid submission and prediction of coding potential (https://wlcb.oit.uci.edu/cpat/). CNCI (Coding-Non-Coding Index) is designed to differentiate between coding and non-coding transcripts (Sun et al., 2013). It employs a sliding window approach (optimized at 150 nt) to scan all six reading frames and calculates an S-score for each window. A dynamic programming algorithm then identifies the Most-Like Coding Sequence (MLCDS) with the highest cumulative S-score. Features derived from the MLCDS are used to train an SVM classifier for final coding potential prediction.

**FIGURE 5:**
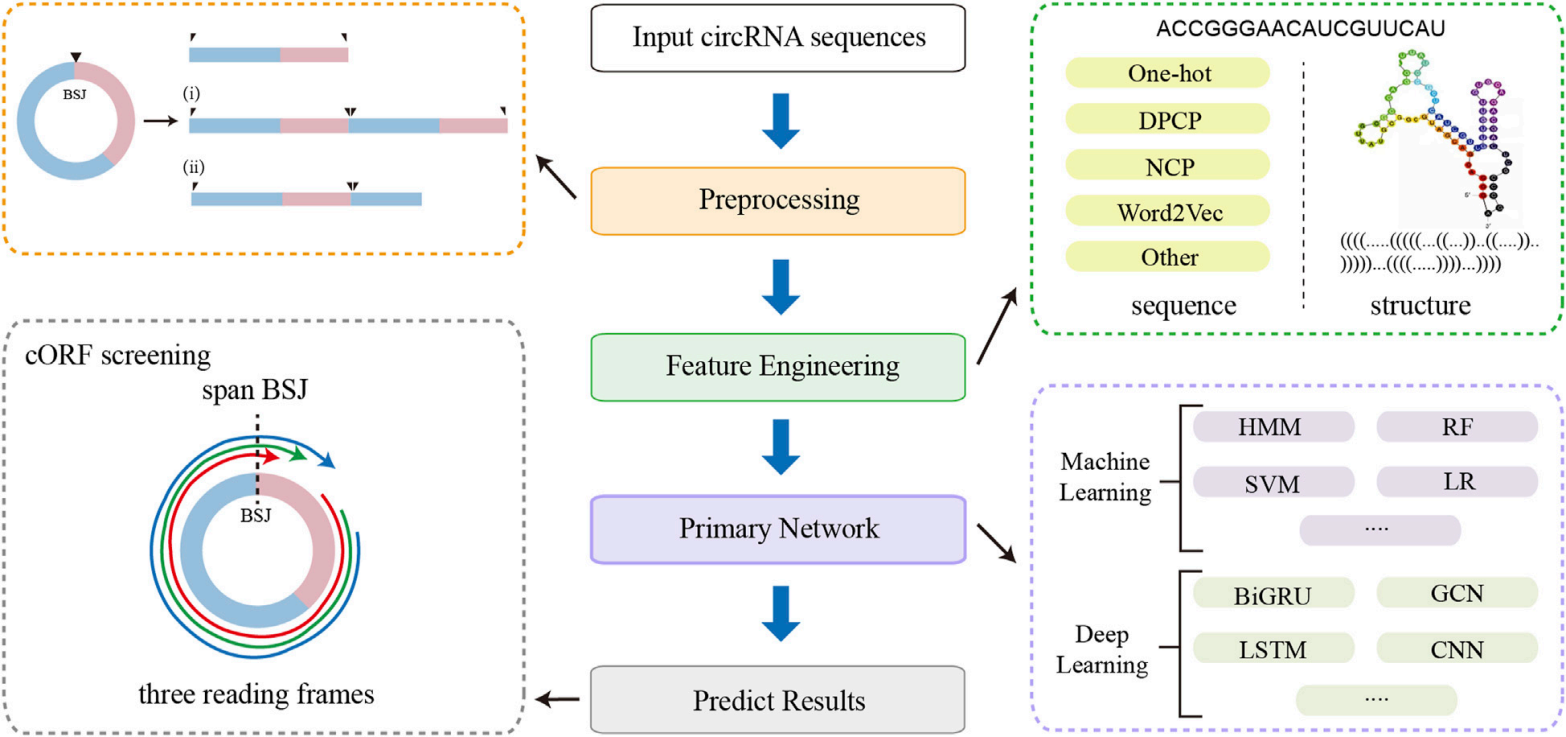
The workflow of linear RNA-based prediction tools adapted for circRNAs, including sequence preprocessing, feature engineering, primary network, and cORF screening.

Since linear RNA-based methods do not inherently account for the covalently closed structure of circRNAs, dedicated sequence preprocessing steps are necessary when adapting these tools for circRNA translation prediction. Figure 5 illustrates the general workflow for this adaptation, including sequence preprocessing, feature engineering, model architecture, and cORF screening. The primary goal of preprocessing is to enable the models can capture sequence features flanking the BSJ, thereby preserving the complete upstream and downstream context of the circular transcript. Common preprocessing strategies include tandemly duplicating the circRNA sequence or appending the 5′ fragment to the 3′ end to simulate circularity in a linear input format. During cORF identification, all potential start codons are considered, and candidate cORFs are required to span the BSJ, providing evidence that the ORF originates from a circular rather than a linear transcript.

## 6 Evaluation of circRNA translation methods

In the previous section, we introduced various tools developed to predict the translational potential of circRNAs. While some of these tools can directly assess whether a circRNA is translated into a protein, others are limited to predicting the presence of translational regulatory features within the sequence. However, a unified benchmark for evaluating the performance of circRNA translation prediction methods is currently lacking.

### 6.1 Datasets and methods

(i) Benchmark Dataset Construction. To systematically evaluate the performance of computational methods in predicting circRNA coding potential, we constructed benchmark datasets from public resources. Positive samples comprised circRNAs with definitive translation evidence, while negative samples consisted of circRNAs lacking any such support. To ensure rigorous evaluation, all selected data were non-overlapping with the training sets used by the assessed methods. Specifically, we sourced positive samples from two origins: a. Experimentally validated translatable circRNAs manually curated from literature in the riboCIRC database; b. Mass spectrometry (MS)-verified translatable human circRNAs reported by Cao and Li (2022). After removing sequence redundancies using CD-HIT (100% identity threshold), we obtained 803 unique positive sequences. Negative samples were selected from the TransCirc database, which catalogs >320,000 circRNAs with systematically annotated translation potential across seven evidence tiers (including IRES elements, m^6^A modifications, and Ribo-seq support). We extracted circRNAs devoid of all translation evidence and applied identical CD-HIT filtering, yielding 661 negative sequences.
(ii) Sequence Preprocessing and ORF Filtering. We evaluated five computational methods: three originally designed for linear RNA translation analysis and two specialized for circRNA translation. To enable comparative assessment within a unified framework, linear RNA methods require sequence preprocessing for circRNA applicability. Specifically, each input circRNA sequence (length n) underwent tandem duplication to generate 3n-length constructs, simulating circular topology. Following ORF prediction, to identify circRNA-specific ORFs, only those spanning the BSJ with an overlap of at least 10 nucleotides were retained. In contrast, circRNA-specific methods processed native sequences without preprocessing and required no post-prediction filtering.
(iii) Computational Implementation Details. All methods were evaluated on a workstation with an Intel Core i7-12700KF CPU (12 cores, 20 threads), 128 GB RAM, running Ubuntu 20.04 LTS. Python environments were configured according to each tool’s requirements.

We evaluated five computational tools for circRNA coding potential prediction. CICADA analysis was performed via its web server with the “Top 5 ranked ORFs” option. cirCodAn utilized the vertebrate-specific model (-m models/VERT_circ) for human circRNAs, reporting only translatable candidates with ORF coordinates. CPAT employed its human logistic regression model with coding/noncoding classification at CP ≥ 0.364 cutoff. CPC2 requires a minimal input FASTA file and an output prefix to generate coding classifications and ORF positions. CNCI implemented its vertebrate model (-m ve), outputting coding labels and ORF coordinates in CNCI. index files.

(iv) Performance Evaluation Metrics. For performance evaluation, we employed classification metrics, including accuracy, precision, recall, and F1-score.

$$Accuracy=\frac{TP+TN}{TN+FP+TP+FN}$$

$$Precision=\frac{TP}{TP+FP}$$

$$Recall=\frac{TP}{TP+FN}$$

$$F1\;score=\frac{2\times Precision\times Recall}{Precision+Recall}$$

### 6.2 Evaluation results

When applying linear RNA-based methods to predict circRNA translation potential, we introduced a filtering step aimed at reducing false-positive predictions. After filtering, all three evaluated linear RNA-based methods exhibited varying degrees of decline in accuracy and F1 scores (Table 3). Prior to filtering, CPAT achieved the highest performance, achieving an accuracy of 87.98% and an F1-score of 89.29%, outperforming other methods. However, after applying the filtering criteria, CPAT’s accuracy and F1-score decreased by 3.35% and 4.01%, respectively, resulting in the loss of its top-ranking status. Moreover, given the stringent requirements specific to circRNA translation prediction, the post-filtering performance metrics of linear RNA-based methods may still represent an overestimation.

**TABLE 3:** Comparison of the performance of linear RNA-based analysis methods prior to and following filtering.

Method	Data Status	Accuracy	Precision	Recall	F1 score
CNCI	Raw	84.90%	95.05%	76.46%	84.75%
	Filtered	80.19%	94.61%	67.75%	78.96%
	Difference	**4.71%**	**0.44%**	**8.72%**	**5.79%**
CPAT	Raw	87.98%	87.28%	91.41%	89.29%
	Filtered	84.63%	89.81%	81.20%	85.28%
	Difference	**3.35%**	**−2.53%**	**10.21%**	**4.01%**
CPC2	Raw	80.40%	96.24%	66.87%	78.91%
	Filtered	79.17%	96.80%	64.13%	77.15%
	Difference	**1.23%**	**−0.57%**	**2.74%**	**1.76%**

**FIGURE 6:**
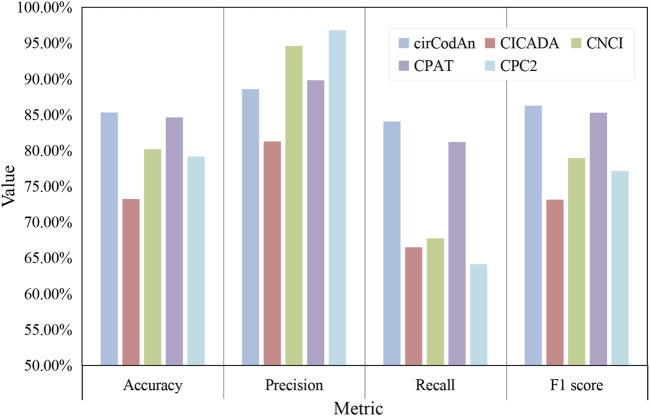
Performance evaluation of circRNA translation analysis methods.

In addition, we systematically compared several computational methods that utilize only circRNA sequences as input, with the results summarized in Figure 6. Among them, cirCodAn consistently demonstrated the best overall performance, achieving the highest accuracy (85.31%), recall (84.06%), and F1-score (86.26%). CPAT ranked second in accuracy, recall, and F1-score, reflecting stable and reliable performance. Notably, CPC2 achieved the highest precision, indicating superior specificity in its predictions. Interestingly, CICADA—despite being specifically developed based on circRNA features—demonstrated relatively lower performance, achieving an accuracy of 73.22% and an F1-score of 73.15%. This discrepancy may stem from the differences in the training data of CICADA. Its positive samples comprised experimentally validated circRNAs from the TransCirc database, while negative samples consisted of intronic sequences. However, the negative set used during evaluation comprised circRNAs from TransCirc lacking experimental evidence of translation; these sequences derived from exonic regions, and they present a greater challenge for accurate discrimination, potentially impairing the model’s predictive performance. Regarding computational efficiency, all methods completed their computations within 3 minutes (Table 4), with CPC2 exhibiting the fastest runtime and CNCI the slowest. Due to its complex deployment requirements, CICADA was run via its online web server, resulting in runtime exceeding 1 day. Taken together, these findings suggest that cirCodAn currently represents the most reliable tool for assessing the translational potential of circRNAs.

**TABLE 4:** Runtime Comparison of circRNA Translation Prediction Methods.

Method	Runtime
cirCodAn	5.191s
CNCI	164.164s
CPAT	3.194s
CPC2	2.464s
CICADA	-

Artificial intelligence technologies have been widely employed in the study of circRNA translation. Initial approaches, primarily based on linear RNA, utilized conventional machine learning algorithms such as SVM and logistic regression. These methods depend on handcrafted features (e.g., k-mer composition, ORF length, or GC content), which are biologically interpretable and directly related to RNA translation mechanisms. With the advancement of computational methods, more recent methods have increasingly incorporated deep learning architectures, such as CNNs, LSTMs, and GCNs. These models automatically learn complex representations from raw sequence and structural data, thereby obviating the need for manual feature engineering. Nonetheless, deep learning methods often face challenges such as reduced interpretability, higher demands for large-scale annotated datasets, and increased computational resource requirements.

Given the limited availability of well-annotated circRNA translation data, traditional machine-learning approaches retain utility for circRNA translation prediction. Tools such as cirCodAn and CNCI exemplify such methods, but due to scarce high-quality circRNA training data coupled with the critical need for prediction accuracy, fine-tuning large language models (LLMs) presents a promising strategy. LLMs are advanced deep learning architectures pretrained on massive datasets, enabling them to capture complex contextual representations and transfer knowledge across tasks (Kim et al., 2023). This facilitates robust performance through fine-tuning even with limited task-specific data. Recently developed RNA-specific LLMs—such as RNA-FM and RNAErnie—provide new opportunities to improve circRNA translation prediction (Shen et al., 2024; Wang et al., 2024). Fine-tuning these large pretrained models may significantly enhance performance in circRNA translation analysis, particularly under data-constrained conditions.

## 7 Concluding remarks

circRNA translation has emerged as a frontier in RNA biology, with increasing evidence revealing its pivotal roles in cellular regulation and disease pathogenesis. Several circRNA-encoded proteins have shown significant promise as biomarkers and therapeutic targets in cancer and other major diseases (Lu et al., 2021; Wen et al., 2022). In this review, we first present a comprehensive overview of circRNA biogenesis, translation mechanisms, and relevant databases that provide the theoretical framework and data foundation for method development. Subsequently, we introduce existing circRNA translation analysis tools along with their underlying methodologies, and conduct a systematic performance comparison of various sequence-based prediction methods using a unified benchmark dataset.

Despite recent advances, key limitations constrain the accuracy and generalizability of circRNA translation prediction. Current bioinformatic tools predominantly rely on high-throughput sequencing data (e.g., Ribo-seq, mass spectrometry), which—while powerful—introduce substantial costs and analytical complexity. Simultaneously, machine learning approaches for direct sequence-based prediction face multifaceted constraints.

A limitation stems from inaccurate full-length circRNA sequences generated through short-read assembly (Huang et al., 2021; Li et al., 2021). Most entries in translational databases derive from short-read assembly, where misassembly sequences may propagate biases into downstream predictions. Long-read sequencing technologies address this limitation by capturing complete circRNA isoforms (Hou et al., 2023), enabling precise identification of BSJ-spanning ORFs and regulatory motifs essential for translation initiation.

The environmental dependency of circRNA translation further complicates predictions: translation occurs exclusively during cellular stress, differentiation, or disease states in many cases. Bulk sequencing methods ignore such heterogeneity by averaging signals across cell populations, masking rare translational events. Integrated single-cell RNA sequencing and proteomics will resolve this by detecting cell-type-specific circRNA-derived peptides with unprecedented resolution (Peng et al., 2023). In addition, low-abundance circRNA peptides often evade detection in conventional bulk MS/Ribo-seq datasets, this approach will provide high-quality training data for predictive models.

The high cost of experimental validation further limits access to high-quality, well-annotated circRNA datasets. In terms of data, *in silico* data augmentation offers a practical solution (Shorten et al., 2021): by exploiting the covalently closed circular topology of circRNAs, the circular sequence can be virtually broken at any position along the loop, generating multiple linear sequence variants of the same molecule. This approach artificially expands training samples for both coding and non-coding circRNAs, enhancing model robustness without incurring experimental costs. In terms of model architecture, transfer learning represents a compelling strategy to enhance model generalizability. In this framework, LLMs can be pre-trained on extensive linear RNA datasets to capture generalizable sequence and structural features, and subsequently fine-tuned on circRNA-specific tasks to exploit shared biological characteristics (Thirunavukarasu et al., 2023). Furthermore, implementing multi-task learning architectures that jointly optimize auxiliary objectives—such as ORF identification or the detection of cis-regulatory elements (e.g., IRES motifs and m6A modification sites)—can promote the learning of robust, shared representations (Yang, 2024). Such approaches have the potential to substantially improve prediction accuracy and model robustness across diverse biological contexts.

In summary, circRNA translation analysis remains challenging in several aspects, including data integration and model optimization. However, with ongoing advancements in computational approaches and experimental technologies, more comprehensive and accurate identification of circRNA translation events is anticipated, facilitating deeper insights into disease mechanisms and broader clinical applications.

## References

[B1] Aktaş T. Avşar Ilık İ. Maticzka D. Bhardwaj V. Pessoa Rodrigues C. Mittler G. (2017). DHX9 suppresses RNA processing defects originating from the alu invasion of the human genome. Nature 544 (7648), 115–119. 10.1038/nature21715 28355180

[B2] Ashwal-Fluss R. Meyer M. Pamudurti N. R. Ivanov A. Bartok O. Hanan M. (2014). circRNA biogenesis competes with pre-mRNA splicing. Mol. Cell 56 (1), 55–66. 10.1016/j.molcel.2014.08.019 25242144

[B3] Barbosa D. F. Oliveira L. S. Kashiwabara A. Y. (2023). “circTIS: a weighted degree string kernel with support vector machine tool for translation initiation sites prediction in circRNA,” in Advances in Bioinformatics and Computational Biology: 16th Brazilian Symposium on Bioinformatics, BSB 2023, Curitiba, Brazil, June 13–16, 2023, 14–24.

[B4] Barbosa D. F. Oliveira L. S. Nachtigall P. G. Valentini Junior R. de Souza N. Paschoal A. R. (2024). cirCodAn: a GHMM-Based tool for accurate prediction of coding regions in circRNA. Adv. Protein Chem. Struct. Biol. 139, 289–334. 10.1016/bs.apcsb.2023.11.012 38448139

[B5] Borao S. Ayté J. Hümmer S. (2021). Evolution of the early spliceosomal complex-from constitutive to regulated splicing. Int. J. Mol. Sci. 22 (22), 12444. 10.3390/ijms222212444 34830325 PMC8624252

[B6] Cao Z. Li G. (2022). MStoCIRC: a powerful tool for downstream analysis of MS/MS data to predict translatable circRNAs. Front. Mol. Biosci. 9, 791797. 10.3389/fmolb.2022.791797 36072432 PMC9441560

[B7] Chang J. Shin M. K. Park J. Hwang H. J. Locker N. Ahn J. (2023). An interaction between eIF4A3 and eIF3g drives the internal initiation of translation. Nucleic Acids Res. 51 (20), 10950–10969. 10.1093/nar/gkad763 37811880 PMC10639049

[B8] Chen C. Y. Sarnow P. (1995). Initiation of protein synthesis by the eukaryotic translational apparatus on circular RNAs. Science 268 (5209), 415–417. 10.1126/science.7536344 7536344

[B9] Chen R. Wang S. K. Belk J. A. Amaya L. Li Z. Cardenas A. (2023). Engineering circular RNA for enhanced protein production. Nat. Biotechnol. 41 (2), 262–272. 10.1038/s41587-022-01393-0 35851375 PMC9931579

[B10] Chen X. Han P. Zhou T. Guo X. Song X. Li Y. (2016). circRNADb: a comprehensive database for human circular RNAs with protein-coding annotations. Sci. Rep. 6, 34985. 10.1038/srep34985 27725737 PMC5057092

[B11] Conn S. J. Pillman K. A. Toubia J. Conn V. M. Salmanidis M. Phillips C. A. (2015). The RNA binding protein quaking regulates formation of circRNAs. Cell 160 (6), 1125–1134. 10.1016/j.cell.2015.02.014 25768908

[B12] Conn V. M. Chinnaiyan A. M. Conn S. J. (2024). Circular RNA in cancer. Nat. Rev. Cancer 24 (9), 597–613. 10.1038/s41568-024-00721-7 39075222

[B13] Drula R. Braicu C. Neagoe I. B. (2024). Current advances in circular RNA detection and investigation methods: are we running in circles? Wiley Interdiscip. Rev. RNA 15 (3), e1850. 10.1002/wrna.1850 38702943

[B14] Dubin R. A. Kazmi M. A. Ostrer H. (1995). Inverted repeats are necessary for circularization of the mouse testis sry transcript. Gene 167 (1-2), 245–248. 10.1016/0378-1119(95)00639-7 8566785

[B15] Fan L. Zhou X. Li M. Gao A. Yu H. Tian H. (2025). CICADA: a circRNA effort toward the ghost proteome. Nucleic Acids Res. 53 (1), gkae1179. 10.1093/nar/gkae1179 39711481 PMC11724281

[B16] Fan X. Yang Y. Chen C. Wang Z. (2022). Pervasive translation of circular RNAs driven by short IRES-Like elements. Nat. Commun. 13 (1), 3751. 10.1038/s41467-022-31327-y 35768398 PMC9242994

[B17] Fang X. Miao R. Wei J. Wu H. Tian J. (2022). Advances in multi-omics study of biomarkers of glycolipid metabolism disorder. Comput. Struct. Biotechnol. J. 20, 5935–5951. 10.1016/j.csbj.2022.10.030 36382190 PMC9646750

[B18] Gao Y. Zhang J. Zhao F. (2018). Circular RNA identification based on multiple seed matching. Brief. Bioinform 19 (5), 803–810. 10.1093/bib/bbx014 28334140

[B19] Gentry R. C. Ide N. A. Comunale V. M. Hartwick E. W. Kinz-Thompson C. D. Gonzalez R. L. Jr. (2025). The mechanism of mRNA cap recognition. Nature 637 (8046), 736–743. 10.1038/s41586-024-08304-0 39663447 PMC12704497

[B20] Godet A. C. David F. Hantelys F. Tatin F. Lacazette E. Garmy-Susini B. (2019). IRES trans-acting factors, key actors of the stress response. Int. J. Mol. Sci. 20 (4), 924. 10.3390/ijms20040924 30791615 PMC6412753

[B21] Guttman M. Russell P. Ingolia N. T. Weissman J. S. Lander E. S. (2013). Ribosome profiling provides evidence that large noncoding RNAs do not encode proteins. Cell 154 (1), 240–251. 10.1016/j.cell.2013.06.009 23810193 PMC3756563

[B22] Hashemi M. Khosroshahi E. M. Daneii P. Hassanpoor A. Eslami M. Koohpar Z. K. (2025). Emerging roles of CircRNA-miRNA networks in cancer development and therapeutic response. Noncoding RNA Res. 10, 98–115. 10.1016/j.ncrna.2024.09.006 39351450 PMC11440256

[B23] Hossain M. T. Reza M. S. Peng Y. Feng S. Wei Y. (2023). Identification of key genes as potential drug targets for gastric cancer. Tsinghua Sci. Technol. 28 (4), 649–664. 10.26599/TST.2022.9010035

[B24] Hou L. Zhang J. Zhao F. (2023). Full-length circular RNA profiling by nanopore sequencing with CIRI-Long. Nat. Protoc. 18 (6), 1795–1813. 10.1038/s41596-023-00815-w 37045995

[B25] Hu F. Peng Y. Chang S. Luo X. Yuan Y. Zhu X. (2022). Vimentin binds to a novel tumor suppressor protein, GSPT1-238aa, encoded by circGSPT1 with a selective encoding priority to halt autophagy in gastric carcinoma. Cancer Lett. 545, 215826. 10.1016/j.canlet.2022.215826 35839920

[B26] Huang W. Ling Y. Zhang S. Xia Q. Cao R. Fan X. (2021). TransCirc: an interactive database for translatable circular RNAs based on multi-omics evidence. Nucleic Acids Res. 49 (D1), D236–d242. 10.1093/nar/gkaa823 33074314 PMC7778967

[B27] Hwang H. J. Kim Y. K. (2024). Molecular mechanisms of circular RNA translation. Exp. Mol. Med. 56 (6), 1272–1280. 10.1038/s12276-024-01220-3 38871818 PMC11263353

[B28] Ingolia N. T. Ghaemmaghami S. Newman J. R. Weissman J. S. (2009). Genome-wide analysis *in vivo* of translation with nucleotide resolution using ribosome profiling. Science 324 (5924), 218–223. 10.1126/science.1168978 19213877 PMC2746483

[B29] Ivanov A. Memczak S. Wyler E. Torti F. Porath H. T. Orejuela M. R. (2015). Analysis of intron sequences reveals hallmarks of circular RNA biogenesis in animals. Cell Rep. 10 (2), 170–177. 10.1016/j.celrep.2014.12.019 25558066

[B30] Jeck W. R. Sorrentino J. A. Wang K. Slevin M. K. Burd C. E. Liu J. (2013). Circular RNAs are abundant, conserved, and associated with ALU repeats. Rna 19 (2), 141–157. 10.1261/rna.035667.112 23249747 PMC3543092

[B31] Kafasla P. Morgner N. Pöyry T. A. Curry S. Robinson C. V. Jackson R. J. (2009). Polypyrimidine tract binding protein stabilizes the encephalomyocarditis virus IRES structure *via* binding multiple sites in a unique orientation. Mol. Cell 34 (5), 556–568. 10.1016/j.molcel.2009.04.015 19524536

[B32] Kang Y. J. Yang D. C. Kong L. Hou M. Meng Y. Q. Wei L. (2017). CPC2: a fast and accurate coding potential calculator based on sequence intrinsic features. Nucleic Acids Res. 45 (W1), W12–w16. 10.1093/nar/gkx428 28521017 PMC5793834

[B33] Keller W. (1984). The RNA lariat: a new ring to the splicing of mRNA precursors. Cell 39 (3 Pt 2), 423–425. 10.1016/0092-8674(84)90449-5 6568879

[B34] Kelly S. Greenman C. Cook P. R. Papantonis A. (2015). Exon skipping is correlated with exon circularization. J. Mol. Biol. 427 (15), 2414–2417. 10.1016/j.jmb.2015.02.018 25728652

[B35] Kim J. K. Chua M. Rickard M. Lorenzo A. (2023). ChatGPT and large language model (LLM) chatbots: the current state of acceptability and a proposal for guidelines on utilization in academic medicine. J. Pediatr. Urol. 19 (5), 598–604. 10.1016/j.jpurol.2023.05.018 37328321

[B36] Kramer M. C. Liang D. Tatomer D. C. Gold B. March Z. M. Cherry S. (2015). Combinatorial control of Drosophila circular RNA expression by intronic repeats, hnRNPs, and SR proteins. Genes Dev. 29 (20), 2168–2182. 10.1101/gad.270421.115 26450910 PMC4617980

[B37] Kristensen L. S. Andersen M. S. Stagsted L. V. W. Ebbesen K. K. Hansen T. B. Kjems J. (2019). The biogenesis, biology and characterization of circular RNAs. Nat. Rev. Genet. 20 (11), 675–691. 10.1038/s41576-019-0158-7 31395983

[B38] Li H. Xie M. Wang Y. Yang L. Xie Z. Wang H. (2021). riboCIRC: a comprehensive database of translatable circRNAs. Genome Biol. 22 (1), 79. 10.1186/s13059-021-02300-7 33685493 PMC7938571

[B39] Lin H. H. Chang C. Y. Huang Y. R. Shen C. H. Wu Y. C. Chang K. L. (2023). Exon junction complex mediates the cap-independent translation of circular RNA. Mol. Cancer Res. 21 (11), 1220–1233. 10.1158/1541-7786.Mcr-22-0877 37527157

[B40] Liu J. Liu T. Wang X. He A. (2017). Circles reshaping the RNA world: from waste to treasure. Mol. Cancer 16 (1), 58. 10.1186/s12943-017-0630-y 28279183 PMC5345220

[B41] Liu L. Lei X. Wang Z. Meng J. Song B. (2025). TransRM: weakly supervised learning of translation-enhancing N6-methyladenosine (m6A) in circular RNAs. Int. J. Biol. Macromol. 306, 141588. 10.1016/j.ijbiomac.2025.141588 40023417

[B42] Lokras A. G. Bobak T. R. Baghel S. S. Sebastiani F. Foged C. (2024). Advances in the design and delivery of RNA vaccines for infectious diseases. Adv. Drug Deliv. Rev. 213, 115419. 10.1016/j.addr.2024.115419 39111358

[B43] Lu Y. Li Z. Lin C. Zhang J. Shen Z. (2021). Translation role of circRNAs in cancers. J. Clin. Lab. Anal. 35 (7), e23866. 10.1002/jcla.23866 34097315 PMC8275004

[B44] Marei S. Maatouk N. AbouHaidar M. Talhouk R. (2025). Developmental regulation of circRNAs in normal and diseased mammary gland: a focus on circRNA-miRNA networks. J. Mammary Gland. Biol. Neoplasia 30 (1), 8. 10.1007/s10911-025-09580-w 40314719 PMC12048424

[B45] Meng X. Chen Q. Zhang P. Chen M. (2017). CircPro: an integrated tool for the identification of circRNAs with protein-coding potential. Bioinformatics 33 (20), 3314–3316. 10.1093/bioinformatics/btx446 29028266

[B46] Meyer K. D. Patil D. P. Zhou J. Zinoviev A. Skabkin M. A. Elemento O. (2015). 5' UTR m(6)A promotes cap-independent translation. Cell 163 (4), 999–1010. 10.1016/j.cell.2015.10.012 26593424 PMC4695625

[B47] Mohanta A. Chakrabarti K. (2021). Dbr1 functions in mRNA processing, intron turnover and human diseases. Biochimie 180, 134–142. 10.1016/j.biochi.2020.10.003 33038423

[B48] Niu M. Wang C. Zhang Z. Zou Q. (2024). A computational model of circRNA-associated diseases based on a graph neural network: prediction and case studies for follow-up experimental validation. BMC Biol. 22 (1), 24. 10.1186/s12915-024-01826-z 38281919 PMC10823650

[B49] Nott A. Le Hir H. Moore M. J. (2004). Splicing enhances translation in Mammalian cells: an additional function of the exon junction complex. Genes Dev. 18 (2), 210–222. 10.1101/gad.1163204 14752011 PMC324426

[B50] O'Leary E. Jiang Y. Kristensen L. S. Hansen T. B. Kjems J. (2025). The therapeutic potential of circular RNAs. Nat. Rev. Genet. 26 (4), 230–244. 10.1038/s41576-024-00806-x 39789148

[B51] Padgett R. A. Konarska M. M. Grabowski P. J. Hardy S. F. Sharp P. A. (1984). Lariat RNA's as intermediates and products in the splicing of messenger RNA precursors. Science 225 (4665), 898–903. 10.1126/science.6206566 6206566

[B52] Panda A. C. Dudekula D. B. Abdelmohsen K. Gorospe M. (2018). Analysis of circular RNAs using the web tool CircInteractome. Methods Mol. Biol. 1724, 43–56. 10.1007/978-1-4939-7562-4_4 29322439 PMC5897125

[B53] Peng J. Li F. Xu X. Hu S. (2023). Single-cell analysis of circRNA using ddPCR. Methods Mol. Biol. 2689, 169–177. 10.1007/978-1-0716-3323-6_13 37430054

[B54] Peng Y. Xu Y. Zhang X. Deng S. Yuan Y. Luo X. (2021). A novel protein AXIN1-295aa encoded by circAXIN1 activates the Wnt/β-catenin signaling pathway to promote gastric cancer progression. Mol. Cancer 20 (1), 158. 10.1186/s12943-021-01457-w 34863211 PMC8642992

[B55] Roberts L. Wieden H. J. (2018). Viruses, IRESs, and a universal translation initiation mechanism. Biotechnol. Genet. Eng. Rev. 34 (1), 60–75. 10.1080/02648725.2018.1471567 29804514

[B56] Robic A. Cerutti C. Kühn C. Faraut T. (2021). Comparative analysis of the circular transcriptome in muscle, liver, and testis in three livestock species. Front. Genet. 12, 665153. 10.3389/fgene.2021.665153 34040640 PMC8141914

[B57] Rodriguez-Martinez A. Young-Baird S. K. (2025). Polysome profiling is an extensible tool for the analysis of bulk protein synthesis, ribosome biogenesis, and the specific steps in translation. Mol. Biol. Cell 36 (4), mr2. 10.1091/mbc.E24-08-0341 40042939 PMC12005114

[B58] Romanelli M. G. Diani E. Lievens P. M. (2013). New insights into functional roles of the polypyrimidine tract-binding protein. Int. J. Mol. Sci. 14 (11), 22906–22932. 10.3390/ijms141122906 24264039 PMC3856098

[B59] Shi Y. Jia X. Xu J. (2020). The new function of circRNA: translation. Clin. Transl. Oncol. 22 (12), 2162–2169. 10.1007/s12094-020-02371-1 32449127

[B98] Shen T. Hu Z. Sun S. Liu D. Liu D. Wong F. Wang J. (2024). Accurate RNA 3D structure prediction using a language model-based deep learning approach. Nat. Methods 21 (12), 2287–2298. 10.1038/s41592-024-02487-0 39572716 PMC11621015

[B60] Shorten C. Khoshgoftaar T. M. Furht B. (2021). Text data augmentation for deep learning. J. Big Data 8 (1), 101. 10.1186/s40537-021-00492-0 34306963 PMC8287113

[B61] Song R. Guo P. Ren X. Zhou L. Li P. Rahman N. A. (2023). A novel polypeptide CAPG-171aa encoded by circCAPG plays a critical role in triple-negative breast cancer. Mol. Cancer 22 (1), 104. 10.1186/s12943-023-01806-x 37408008 PMC10320902

[B62] Stoll L. Rodríguez-Trejo A. Guay C. Brozzi F. Bayazit M. B. Gattesco S. (2020). A circular RNA generated from an intron of the insulin gene controls insulin secretion. Nat. Commun. 11 (1), 5611. 10.1038/s41467-020-19381-w 33154349 PMC7644714

[B63] Sun L. Luo H. Bu D. Zhao G. Yu K. Zhang C. (2013). Utilizing sequence intrinsic composition to classify protein-coding and long non-coding transcripts. Nucleic Acids Res. 41 (17), e166. 10.1093/nar/gkt646 23892401 PMC3783192

[B64] Sun P. Li G. (2019). CircCode: a powerful tool for identifying circRNA coding ability. Front. Genet. 10, 981. 10.3389/fgene.2019.00981 31649739 PMC6795751

[B65] Surono A. Takeshima Y. Wibawa T. Ikezawa M. Nonaka I. Matsuo M. (1999). Circular dystrophin RNAs consisting of exons that were skipped by alternative splicing. Hum. Mol. Genet. 8 (3), 493–500. 10.1093/hmg/8.3.493 9949208

[B66] Talhouarne G. J. Gall J. G. (2014). Lariat intronic RNAs in the cytoplasm of Xenopus tropicalis oocytes. Rna 20 (9), 1476–1487. 10.1261/rna.045781.114 25051970 PMC4138330

[B67] Thirunavukarasu A. J. Ting D. S. J. Elangovan K. Gutierrez L. Tan T. F. Ting D. S. W. (2023). Large language models in medicine. Nat. Med. 29 (8), 1930–1940. 10.1038/s41591-023-02448-8 37460753

[B99] Wang N. Bian J. Li Y. Li X. Mumtaz S. Kong L. (2024). Multi-purpose RNA language modelling with motif-aware pretraining and type-guided fine-tuning. Nat. Mach. Intelli. 6 (5), 548–557. 10.1038/s42256-024-00836-4

[B68] Wang L. Park H. J. Dasari S. Wang S. Kocher J. P. Li W. (2013). CPAT: coding-potential assessment tool using an alignment-free logistic regression model. Nucleic Acids Res. 41 (6), e74. 10.1093/nar/gkt006 23335781 PMC3616698

[B69] Wang Z. (2018). Diverse roles of regulatory non-coding RNAs. J. Mol. Cell Biol. 10 (2), 91–92. 10.1093/jmcb/mjy026 29762766

[B70] Wei H. Y. Fan X. J. Mao M. W. (2025). A review on circular RNA translation and its implications in disease. Methods Mol. Biol. 2883, 109–137. 10.1007/978-1-0716-4290-0_5 39702706

[B71] Wen S. Y. Qadir J. Yang B. B. (2022). Circular RNA translation: novel protein isoforms and clinical significance. Trends Mol. Med. 28 (5), 405–420. 10.1016/j.molmed.2022.03.003 35379558

[B72] Wesselhoeft R. A. Kowalski P. S. Anderson D. G. (2018). Engineering circular RNA for potent and stable translation in eukaryotic cells. Nat. Commun. 9 (1), 2629. 10.1038/s41467-018-05096-6 29980667 PMC6035260

[B73] Wu W. Zhao F. Zhang J. (2024). circAtlas 3.0: a gateway to 3 million curated vertebrate circular RNAs based on a standardized nomenclature scheme. Nucleic Acids Res. 52 (D1), D52–d60. 10.1093/nar/gkad770 37739414 PMC10767913

[B74] Xiong L. Liu H. S. Zhou C. Yang X. Huang L. Jie H. Q. (2023). A novel protein encoded by circINSIG1 reprograms cholesterol metabolism by promoting the ubiquitin-dependent degradation of INSIG1 in colorectal cancer. Mol. Cancer 22 (1), 72. 10.1186/s12943-023-01773-3 37087475 PMC10122405

[B75] Yang J. (2024). Multi-task learning for medical foundation models. Nat. Comput. Sci. 4 (7), 473–474. 10.1038/s43588-024-00658-9 39030385

[B76] Yang Y. Fan X. Mao M. Song X. Wu P. Zhang Y. (2017). Extensive translation of circular RNAs driven by N(6)-methyladenosine. Cell Res. 27 (5), 626–641. 10.1038/cr.2017.31 28281539 PMC5520850

[B77] Yang Y. Wang Z. (2019). IRES-Mediated cap-independent translation, a path leading to hidden proteome. J. Mol. Cell Biol. 11 (10), 911–919. 10.1093/jmcb/mjz091 31504667 PMC6884710

[B78] Yi Q. Feng J. Lan W. Shi H. Sun W. Sun W. (2024). CircRNA and lncRNA-encoded peptide in diseases, an update review. Mol. Cancer 23 (1), 214. 10.1186/s12943-024-02131-7 39343883 PMC11441268

[B79] Yoshimoto R. Kataoka N. Okawa K. Ohno M. (2009). Isolation and characterization of post-splicing lariat-intron complexes. Nucleic Acids Res. 37 (3), 891–902. 10.1093/nar/gkn1002 19103666 PMC2647322

[B80] Yuan L. Zhao L. Lai J. Jiang Y. Zhang Q. Shen Z. (2024). iCRBP-LKHA: large convolutional kernel and hybrid channel-spatial attention for identifying circRNA-RBP interaction sites. PLoS Comput. Biol. 20 (8), e1012399. 10.1371/journal.pcbi.1012399 39173070 PMC11373821

[B81] Zang X. He X. Y. Xiao C. M. Lin Q. Wang M. Y. Liu C. Y. (2024). Circular RNA-Encoded oncogenic PIAS1 variant blocks immunogenic ferroptosis by modulating the balance between SUMOylation and phosphorylation of STAT1. Mol. Cancer 23 (1), 207. 10.1186/s12943-024-02124-6 39334380 PMC11438063

[B82] Zaphiropoulos P. G. (1997). Exon skipping and circular RNA formation in transcripts of the human cytochrome P-450 2C18 gene in epidermis and of the rat androgen binding protein gene in testis. Mol. Cell Biol. 17 (6), 2985–2993. 10.1128/mcb.17.6.2985 9154796 PMC232150

[B83] Zhang G. Hou J. Mei C. Wang X. Wang Y. Wang K. (2023a). Effect of circular RNAs and N6-methyladenosine (m6A) modification on cancer biology. Biomed. Pharmacother. 159, 114260. 10.1016/j.biopha.2023.114260 36657303

[B84] Zhang J. Zhang H. Ju Z. Peng Y. Pan Y. Xi W. (2023b). JCcirc: circRNA full-length sequence assembly through integrated junction contigs. Brief. Bioinform 24 (6), bbad363. 10.1093/bib/bbad363 37833842

[B85] Zhang L. Hou C. Chen C. Guo Y. Yuan W. Yin D. (2020a). The role of N(6)-methyladenosine (m(6)A) modification in the regulation of circRNAs. Mol. Cancer 19 (1), 105. 10.1186/s12943-020-01224-3 32522202 PMC7285594

[B86] Zhang P. Dai M. (2022). CircRNA: a rising star in plant biology. J. Genet. Genomics 49 (12), 1081–1092. 10.1016/j.jgg.2022.05.004 35644325

[B87] Zhang X. Peng Y. Huang Y. Yang M. Yan R. Zhao Y. (2018). SMG-1 inhibition by miR-192/-215 causes epithelial-mesenchymal transition in gastric carcinogenesis *via* activation of wnt signaling. Cancer Med. 7 (1), 146–156. 10.1002/cam4.1237 29239144 PMC5773975

[B88] Zhang X. Peng Y. Yuan Y. Gao Y. Hu F. Wang J. (2020b). Histone methyltransferase SET8 is regulated by miR-192/215 and induces oncogene-induced senescence *via* p53-dependent DNA damage in human gastric carcinoma cells. Cell Death Dis. 11 (10), 937. 10.1038/s41419-020-03130-4 33127874 PMC7599338

[B89] Zhang X. Zou Q. Niu M. Wang C. (2025). Predicting circRNA-disease associations with shared units and multi-channel attention mechanisms. Bioinformatics 41 (3), btaf088. 10.1093/bioinformatics/btaf088 40045181 PMC11919450

[B90] Zhang Y. Qi W. Wu Y. (2023c). EIF4A3-induced circular RNA SCAP facilitates tumorigenesis and progression of non-small-cell lung cancer *via* miR-7/SMAD2 signaling. Environ. Sci. Pollut. Res. Int. 30 (24), 65237–65249. 10.1007/s11356-023-26307-8 37079240 PMC10182944

[B91] Zhang Y. Zhang X. O. Chen T. Xiang J. F. Yin Q. F. Xing Y. H. (2013). Circular intronic long noncoding RNAs. Mol. Cell 51 (6), 792–806. 10.1016/j.molcel.2013.08.017 24035497

[B92] Zhang Z. Fu Y. Ju X. Zhang F. Zhang P. He M. (2024). Advances in engineering circular RNA vaccines. Pathogens 13 (8), 692. 10.3390/pathogens13080692 39204292 PMC11356823

[B93] Zhao J. Wu J. Xu T. Yang Q. He J. Song X. (2018). IRESfinder: identifying RNA internal ribosome entry site in eukaryotic cell using framed k-mer features. J. Genet. Genomics 45 (7), 403–406. 10.1016/j.jgg.2018.07.006 30054216

[B94] Zhong S. Feng J. (2022). CircPrimer 2.0: a software for annotating circRNAs and predicting translation potential of circRNAs. BMC Bioinforma. 23 (1), 215. 10.1186/s12859-022-04705-y PMC9169404 35668371

[B95] Zhou W. Y. Cai Z. R. Liu J. Wang D. S. Ju H. Q. Xu R. H. (2020). Circular RNA: metabolism, functions and interactions with proteins. Mol. Cancer 19 (1), 172. 10.1186/s12943-020-01286-3 33317550 PMC7734744

[B96] Zhou Y. Wu J. Yao S. Xu Y. Zhao W. Tong Y. (2023). DeepCIP: a multimodal deep learning method for the prediction of internal ribosome entry sites of circRNAs. Comput. Biol. Med. 164, 107288. 10.1016/j.compbiomed.2023.107288 37542919

[B97] Zuo Y. Liu W. Jin Y. Pan Y. Fan T. Fu X. (2024). C2CDB: an advanced platform integrating comprehensive information and analysis tools of cancer-related circRNAs. Bioinform Adv. 4 (1), vbae112. 10.1093/bioadv/vbae112 39246384 PMC11379471

